# Cholera Vaccination Campaign Contributes to Improved Knowledge Regarding Cholera and Improved Practice Relevant to Waterborne Disease in Rural Haiti

**DOI:** 10.1371/journal.pntd.0002576

**Published:** 2013-11-21

**Authors:** Omowunmi Aibana, Molly Franke, Jessica Teng, Johanne Hilaire, Max Raymond, Louise C. Ivers

**Affiliations:** 1 Department of Medicine, Brigham and Women's Hospital, Boston, Massachusetts, United States of America; 2 Department of Global Health and Social Medicine, Harvard Medical School, Boston, Massachusetts, United States of America; 3 Division of Global Health Equity, Brigham and Women's Hospital, Boston, Massachusetts, United States of America; 4 Partners In Health - Boston, Boston, Massachusetts, United States of America; 5 Partners In Health - Zanmi Lasante, Pont-Tambour, Impasse Guillaume #6, St Marc, Haiti; Massachusetts General Hospital, United States of America

## Abstract

**Background:**

Haiti's cholera epidemic has been devastating partly due to underlying weak infrastructure and limited clean water and sanitation. A comprehensive approach to cholera control is crucial, yet some have argued that oral cholera vaccination (OCV) might result in reduced hygiene practice among recipients. We evaluated the impact of an OCV campaign on knowledge and health practice in rural Haiti.

**Methodology/Principal Findings:**

We administered baseline surveys on knowledge and practice relevant to cholera and waterborne disease to every 10th household during a census in rural Haiti in February 2012 (N = 811). An OCV campaign occurred from May–June 2012 after which we administered identical surveys to 518 households randomly chosen from the same region in September 2012. We compared responses pre- and post-OCV campaign.

Post-vaccination, there was improved knowledge with significant increase in percentage of respondents with ≥3 correct responses on cholera transmission mechanisms (odds ratio[OR] 1.91; 95% confidence interval[CI] 1.52–2.40), preventive methods (OR 1.83; 95% CI 1.46–2.30), and water treatment modalities (OR 2.75; 95% CI 2.16–3.50). Relative to pre-vaccination, participants were more likely post-OCV to report always treating water (OR 1.62; 95% CI 1.28–2.05). Respondents were also more likely to report hand washing with soap and water >4 times daily post-vaccine (OR 1.30; 95% CI 1.03–1.64). Knowledge of treating water as a cholera prevention measure was associated with practice of always treating water (OR 1.47; 95% CI 1.14–1.89). Post-vaccination, knowledge was associated with frequent hand washing (OR 2.47; 95% CI 1.35–4.51).

**Conclusion:**

An OCV campaign in rural Haiti was associated with significant improvement in cholera knowledge and practices related to waterborne disease. OCV can be part of comprehensive cholera control and reinforce, not detract from, other control efforts in Haiti.

## Author Summary

In October 2010, Haiti experienced a cholera outbreak that is now considered one of the largest cholera epidemics in recent history. A comprehensive approach is necessary to successfully fight the epidemic and proven methods for controlling cholera include improving access to clean water and sanitation as well as widespread hygiene education. In addition, there are two safe cholera vaccines approved for use. The authors conducted surveys before and after a cholera vaccination campaign, that included a public health educational component, in rural Haiti; surveys addressed knowledge of cholera and hygiene practices such as hand washing and water treatment, which are crucial for preventing waterborne diseases such as cholera. The authors found that after the vaccination campaign, knowledge of cholera improved significantly. There was also significant increase in reported hand washing and water treatment post vaccination. Furthermore, there was an association between knowledge and hygiene practices. Therefore, this study demonstrates that cholera vaccination can be a complementary tool in the fight against cholera in Haiti and will not detract from other control efforts.

## Introduction

In October 2010, a cholera outbreak began in the Artibonite and Centre Departments of Haiti [1]. By December, cholera had been identified in all 10 departments of Haiti and has since reached neighboring countries [2], [3]. Cholera is an acute, watery diarrheal infection caused by the bacterium *Vibrio cholerae* of the O1 or O139 serogroup; and it can rapidly lead to severe dehydration and death if untreated. However, effective therapy can decrease mortality rate from more than 50% to less than 0.2% [4].

Efforts to control the cholera outbreak have been hampered by weak health systems and lack of clean water and adequate sanitation in Haiti. In 2008, only 17% of Haiti's population used improved sanitation facilities while 12% had access to piped, treated water [5]. In addition, conditions in Haiti further deteriorated on January 12, 2010 when the country suffered a devastating 7.0-magnitude earthquake that killed thousands and rendered approximately 2 million individuals homeless [6]. Pockets of densely populated areas resulting from internal migration after the earthquake likely contributed to an explosive outbreak in Haiti. Rural areas and urban slums were particularly vulnerable to the rapid spread of a waterborne disease such as cholera. Furthermore, Haiti's population had no prior exposure or immunity to *V. cholerae* [7]. Moreover, analysis of the *V. cholerae* strain in Haiti revealed a variant strain (serotype Ogawa, biotype El Tor) known to be associated with more severe illness [8], [9]. Between October 2010 and May 2013, there were over 600,000 cases of infection and more than 8,000 cholera deaths reported [10]. In 2011, the cholera epidemic in Haiti accounted for 58% of all cholera cases and 37% of all cholera deaths reported to the World Health Organization (WHO) [11].

A comprehensive approach is necessary to fight the cholera epidemic in Haiti and proven cholera control measures include: active case finding, improving water and sanitation, and widespread hygiene education [12]–[14]. In addition, there are two safe oral cholera vaccines (OCV), approved by the WHO for use in cholera endemic areas [15]. Some have argued that cholera vaccination might detract from other prevention efforts and result in diminished hygiene practices among vaccine recipients [16]–[18]. Yet, there is no evidence indicating that cholera vaccination reduces hygiene practice.

Knowledge, Attitude, and Practice (KAP) surveys have been used in various settings to assess existing knowledge and hygiene practices relevant to prevention and transmission of diarrheal diseases, including cholera [19]–[22]. KAP surveys have also been employed in areas of cholera outbreak to measure uptake of knowledge and behavioral changes in response to educational activities aimed at cholera control [23], [24]. In December 2010, a KAP survey was conducted in resource-limited communities of Port-au-Prince, Haiti to assess the effectiveness of public health campaigns on cholera education [24]. The study showed high knowledge of cholera signs and transmission mechanisms as well as improvement in water treatment practices after the outbreak. However, there have been no studies evaluating the effect of vaccination campaigns for waterborne, diarrheal diseases on knowledge and practices related to these diseases.

We aimed to assess the impact of an OCV campaign on knowledge of cholera and health practice related to waterborne illness in rural Haiti. We hypothesized that the campaign, which had been implemented with an educational component, would lead to improved knowledge and behavior critical for cholera control and therefore had served to bolster efforts in the fight against cholera in Haiti.

## Methods

Ethics Statement: Data were collected as part of a public health campaign; therefore informed consent was not required from survey respondents. Institutional Review Board approval was received from Partners Healthcare for post-hoc analysis of the de-identified dataset.

We analyzed data from the rural 5^th^ section of St. Marc, also known as Bocozel (Figure S1), in the Artibonite Department of Haiti, where between May and June 2012, the non-profit organization, Partners In Health, carried out a pilot OCV campaign in support of the Haitian Ministry of Health [25]. In February 2012, prior to vaccine implementation, a census was undertaken in Bocozel, resulting in enumeration of 9,517 households. Empty households were visited twice, and if neighboring households could not provide information to confirm that a third visit was warranted, the household was not counted in the census. During the census, every 10^th^ household was invited to participate in a baseline survey on knowledge and practices regarding cholera and waterborne disease.

The survey gathered information on sociodemographic characteristics; knowledge about means of cholera transmission, preventive measures, and water treatment modalities; practices related to frequency of water treatment and hand washing; type of toilet access; and source of drinking water. Knowledge questions prompted respondents to provide as many answers as they could to the following questions: “How can a person get cholera?” “What can you do to avoid getting cholera?” and “What are the methods of treating water that you drink?” Examples of appropriate responses for cholera transmission mechanisms included: “drinking untreated water,” “eating uncooked food,” and “dirty hands.” For cholera prevention methods, suitable answers included: “treat water,” “eat cooked food, and “wash hands.” For hygiene practices, respondents were asked to choose the option that described their frequency of water treatment among: “always,” “almost always,” “often,” “sometimes,” and “almost never.” Respondents were also asked to report the number of times they washed their hands with soap and water daily. Knowledge questions were directed to the individual responding, and practice questions were related to the household. Trained enumerators (locally recruited Haitians who had completed high school) administered surveys to one adult individual (male or female, ≥18 years) identified by members of the household as the head or, in the absence of head of household, a representative of the household. Enumerators received a 2-day training on the use of hardware and software used for data collection as well as the survey modules. Refresher trainings were conducted prior to the administration of each vaccine dose.

The OCV campaign was executed in 2 phases with individuals aged 10 years and above targeted in the first phase, and children between the ages of 1 and 10 years targeted in the second phase. The campaign is described in detail elsewhere [25]. Prior to the campaign, meetings with key stakeholders, community focus groups, and Ministry of Health representatives led to the generation of key messages about cholera prevention and cholera vaccine that were used as part of the vaccination campaign (Table S1). Before and throughout the period of vaccination, educational information was disseminated verbally via radio shows, sound trucks, town criers, local television and was printed on T-shirts and posters. Members of the vaccination team were encouraged to share education messages at every contact with the public. These messages were also communicated by enumerators to household members in the census, after all data collection was complete. Education information was thus provided directly to at least one representative of all enumerated households. All vaccine recipients received the same information during vaccination days, and the entire community received information during the period of the campaign. Printed educational information was not a major focus of the campaign because of low literacy rates in the region.

In September 2012, after the vaccine campaign, a follow-up survey was conducted to estimate vaccination coverage, and as a secondary objective, to evaluate knowledge and practice about cholera. De-identification of pre-vaccine survey data precluded resurveying the same participants; therefore, a list of 600 households was randomly generated from the 9,517 households enumerated during the census using a random number generator in Microsoft Excel. The same survey tool used in the pre-vaccination phase was administered to these households in addition to questions about receipt of cholera vaccine. The same enumerators collected census data and conducted both surveys with the exception of a few staff who were not available at the second time point.

We analyzed results from both surveys using Statistical Analysis System (SAS 9.3). Chi-square and Wilcoxon rank-sum tests were used to compare knowledge and practice variables from the pre- and post-vaccination surveys. We used multivariable logistic regression analysis to (1) evaluate changes in knowledge of cholera prevention and transmission and hygiene practices after the vaccine campaign; (2) examine whether proxies for socioeconomic status (i.e. ever having attended school and access to electricity at home) were associated with these outcomes; and (3) assess whether cholera knowledge was associated with hygiene practices. Multivariable models included a variable for survey (1 versus 2), ever having attended school, and electricity access in the home. To assess for confounding, we first identified baseline variables that were differentially distributed between the two surveys and were associated with any outcome at a p-value≤0.05. These variables (farming occupation, latrine, open defecation) were then included in the multivariable models and those that altered the effect estimate for the survey variable by >10% were retained in the final model.

## Results

A total of 811 households from 53 different localities were surveyed pre-vaccination (Survey1), and 518 households from 47 localities were interviewed post-vaccination (Survey2). Eighty-two of the 600 households randomly selected to complete Survey2 (13.7%) were not interviewed: 43 households had been destroyed or no longer existed, 12 households were empty despite two visit attempts, and 1 household resident was deceased. The remaining 26 households were either not accessible because of challenges presented by the rainy season or they could not be physically located based on the information in the census. Because there were few official addresses in this area, drawn markings had been made during the census to label and number houses; and in some cases, they were no longer legible.

### Vaccine coverage

Vaccine coverage is described in detail elsewhere and was estimated between 76.7–92.7% of the population of the region, with the lower limit of the range estimated by census and registration data and the upper limit estimated from Survey2 [25]. A total of 41,242 individuals received 2-dose series of the OCV. Of the 518 Survey2 respondents, 480 (92.7%) [95% CI 90.1%–94.6%] reported receipt of at least one dose of the cholera vaccine, and 419 (80.8%) [95% CI 77.3%–84.0%] provided their vaccination cards for verification.

### Demographic characteristics of survey respondents

Baseline demographic characteristics for pre-and post-vaccine survey respondents were generally similar (Table 1); however statistically significant differences between the two time points were observed for household size, number of people sharing a toilet, toilet type, and having a farming occupation. 65.2% of Survey1 respondents reported use of latrine compared to 46.9% in Survey2. Farming was the most common occupation representing 69.5% of Survey1 respondents and 76.1% in Survey2.

**Table 1:** Characteristics of respondents in household surveys before and after an oral cholera vaccination campaign in rural Haiti, 2012.

	Survey1 (N = 811) February 2012 n (%) or median (IQR)	N* (Survey1)	Survey2 (N = 518) September 2012 n (%) or median (IQR)	N* (Survey2)	p value†
**Household Size (number of people)**	3 (2–5)	811	4 (3–6)	518	<0.001
**Ever Attended School**	393 (48.7)	807	242 (46.9)	516	0.54
**Level of School (among those who ever attended school)**		392		242	0.42
Some Primary School	253 (64.5)		155 (64.1)		
Some Secondary School	105 (26.8)		73 (30.2)		
Basic Literacy Program (not primary school)	28 (7.1)		13 (5.4)		
Beyond Secondary School	6 (1.5)		1 (0.4)		
**Have Electricity**	127 (15.8)	806	79 (15.3)	518	0.82
**Purchase Any Water**	363 (45.2)	804	255 (49.2)	518	0.16
**Farmer**	562 (69.5)	809	394 (76.1)	518	0.01
**Floor Type**		808		518	0.54
Earth	560 (69.3)		369 (71.2)		
Cement	244 (30.2)		149 (28.8)		
Wood	2 (0.3)		0 (0.0)		
Other	2 (0.3)		0 (0.0)		
**Toilet Type**		808		518	<0.0001
Latrine	527 (65.2)		243 (46.9)		
Open Defecation	241 (29.8)		251 (48.5)		
Non-flush Toilet	19 (2.4)		23 (4.4)		
Flush Toilet	9 (1.1)		1 (0.19)		
Other	12 (1.5)		0 (0.0)		
**No. of People Sharing Toilet (among those who did not report use of open defecation)**	9 (5–20)	553	10 (6–23)	267	0.005
**Have Children ≥1 and <5 years in Household**	341 (43.6)	782	221 (42.7)	518	0.78

* Total number of respondents from each Survey with data for the corresponding variable.

† Wilcoxon rank-sum p-values provided for continuous variables; Chi-squared p-values provided for binary variables.

### Comparison of cholera knowledge pre- and post-vaccination campaign

**Figure 1:**
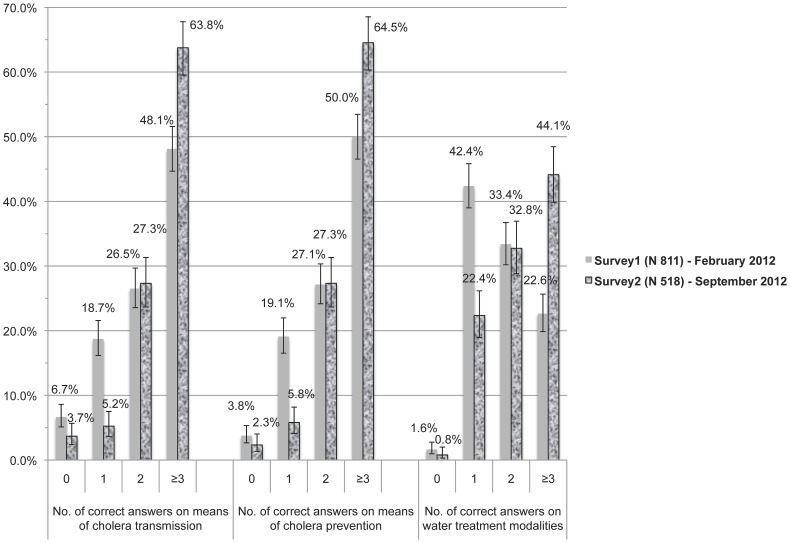
Comparison of cholera knowledge pre- and post-vaccination campaign. Distribution of correct answers for each knowledge question before and after an oral cholera vaccination campaign in rural Haiti, 2012.

Nearly all respondents pre-vaccine (99.1%) and post-vaccine (99.6%) had heard of cholera. A high level of knowledge was defined as greater than the median number of correct answers in Survey1 (Table 2). A significantly higher proportion of Survey2 respondents (63.8%) knew ≥3 correct modes of cholera transmission compared to 48.1% in Survey1 (p<0.0001). A similar pattern was observed with cholera prevention questions. Pre-vaccination, 50.0% of respondents provided ≥3 correct answers on how to avoid cholera compared to 64.5% post-vaccine (p<0.0001). Finally, a higher percentage of individuals in Survey2 (44.1%) knew ≥3 means of water treatment compared to Survey1 (22.6%) with p<0.0001 (Figure 1).

**Table 2:** Prevalence of cholera knowledge and practices of water treatment and hand washing before and after an oral cholera vaccination campaign in rural Haiti, 2012.

Outcome	Survey1 (N = 811) February 2012 n (%) or median (IQR)	N* (Survey1)	Survey2 (N = 518) September 2012 n (%) or median (IQR)	N* (Survey2)	p value†
**Median number of correct answers on means of cholera transmission**	2 (1–3)	796	3 (2–3)	516	<0.0001
**≥3 correct answers on means of cholera transmission**	383 (48.1)	796	329 (63.8)	516	<0.0001
**Median number of correct answers on means of avoiding cholera**	2.5 (2–3)	796	3 (2–3)	516	<0.0001
**≥3 correct answers on means of avoiding cholera**	398 (50.0)	796	333 (64.5)	516	<0.0001
**Median number of correct answers on means of treating water**	2 (1–2)		2 (2–3)		<0.0001
**≥3 correct answers on means of treating water**	181 (22.6)	800	225 (44.1)	510	<0.0001
**ALWAYS treat water**	399 (49.4)	807	321 (62.0)	518	<0.0001
**Wash hands with soap and water >4 times a day**	332 (41.1)	808	242 (46.7)	518	0.05

* Total number of respondents from each Survey with data for the corresponding variable.

† Wilcoxon rank-sum p-values provided for continuous variables; Chi-squared p-values provided for binary variables.

None of the differentially distributed baseline variables significantly changed the effect estimates for any outcome; therefore, only the socioeconomic proxy variables (ever having attended school and access to electricity at home), and no additional variables, were included as covariates in the final multivariable models. For cholera knowledge, post-vaccination surveys were associated with a statistically significant increase in the odds of providing at least 3 correct responses on means of cholera transmission (odds ratio [OR] 1.91; 95% CI 1.52–2.40; p<0.0001). For cholera prevention measures, the odds ratio of knowing 3 or more correct answers in Survey2 compared to Survey1 was 1.83 (95% CI, 1.46–2.30; p<0.0001). Similarly, there was also greater odds of knowing ≥3 ways to treat water in Survey2 relative to Survey1 (OR 2.75; 95% CI, 2.16–3.50; p<0.0001). Ever having attended school and electricity access in the home, were not generally associated with increased knowledge (Table 3); however, we did observe a positive relationship between access to electricity in the home and knowing 3 or more means of avoiding cholera of borderline statistical significance (OR: 1.37; 95% CI: 1.00–1.89).

**Table 3:** Multivariable analyses of factors associated with cholera knowledge and practices of water treatment and hand washing before and after an oral cholera vaccination campaign, February 2012 and September 2012, respectively in rural Haiti.

≥3 correct answers on means of cholera transmission		ODDS RATIO (95% CI)	p value
	Ever attended school	1.00 (0.79–1.25)	0.97
	Access to electricity at home	1.10 (0.81–1.51)	0.54
	Survey2	1.91 (1.52–2.40)	<.0001
**≥3 correct answers on means of avoiding cholera**			
	Ever attended school	0.94 (0.75–1.19)	0.61
	Access to electricity at home	1.37 (1.00–1.89)	0.05
	Survey2	1.83 (1.46–2.30)	<.0001
**≥3 correct answers on means of treating water**			
	Ever attended school	1.07 (0.83–1.37)	0.61
	Access to electricity at home	0.94 (0.66–1.32)	0.70
	Survey2	2.75 (2.16–3.50)	<.0001
**ALWAYS treat water**			
	Ever attended school	1.75 (1.39–2.20)	<.0001
	Access to electricity at home	1.58 (1.13–2.20)	0.01
	Survey2	1.62 (1.28–2.05)	<.0001
**Wash hands with soap and water >4 times a day**			
	Ever attended school	1.70 (1.35–2.15)	<.0001
	Access to electricity at home	1.83 (1.34–2.52)	0.0002
	Survey2	1.30 (1.03–1.64)	0.03

### Comparison of hygiene practices pre- and post-vaccination campaign

The percentage of respondents who reported “always” treating their water increased from 49.4% in Survey1 to 62.0% in Survey2 (p<0.0001). The most common reasons provided for not always treating water were related to access to products. 35.9% had “no products” in Survey1 and 49.2% reported the same reason in Survey2. Products were “hard to get” for 28.2% and 35.0% of respondents in Survey1 and Survey2 respectively. Regarding hand washing practices, 46.7% of Survey2 respondents reported hand washing with soap and water >4 times a day compared to 41.1% in Survey1 (p 0.05). We observed decreased use of river water in Survey2 (42.7%) versus Survey1 (48.0%), although this was not statistically significant (p 0.06).

Multivariable regression analysis of hygiene practice revealed that relative to the pre-vaccination period, post-vaccination participants were more likely to report always treating water (OR1.62; 95% CI, 1.28–2.05; p<0.0001). Similarly, odds of washing hands with soap and water >4 times a day was increased in Survey2 relative to Survey1 (OR1.30; 95% CI, 1.03–1.64; p 0.03). Higher socioeconomic status, as measured by ever having attended school and access to electricity, was associated with increased odds of always treating water and hand washing with soap and water >4 times a day (Table 3). There were no confounding variables associated with practice questions.

### Link between knowledge and practice

Knowledge of water treatment as a means of preventing cholera was associated with the practice of always treating water (OR 1.47; 95% CI, 1.14–1.89; p 0.003). Overall, there was no statistically significant association between knowledge of hand washing as a cholera preventive measure and practice of frequent hand washing (OR 1.10; 95% CI, 0.82–1.46; p 0.53). However, in stratified analyses, knowledge of hand washing as a preventive measure was significantly associated with the practice of washing hands >4 times a day post-vaccine (OR 2.47; 95% CI 1.35–4.51; p 0.003) but not pre-vaccine (OR 0.85; CI 0.61–1.19; p 0.35).

## Discussion

This study aimed to evaluate the impact of a cholera vaccination campaign in rural Haiti on knowledge of cholera and health practice related to transmission and prevention of waterborne illnesses. It revealed that post-vaccination campaign, there was a significant increase in baseline knowledge and improvement in practice essential to cholera control.

Our pre-vaccination surveys revealed that at baseline, 48.1%, 50%, and 22.6% of respondents knew at least 3 means of cholera transmission, prevention methods, and treating water respectively. Nationwide health education campaigns on cholera prevention and transmission seem therefore to have reached this rural community, although, these proportions appear low. This may partly be related to the timing of our study that occurred almost two years after the outbreak when the intensity of public health messaging may have waned. Furthermore, the remote location of our rural study population combined with limited electricity may have hampered access to national mass media campaigns. A KAP survey conducted two months after the onset of cholera in the capital city, Port-au-Prince, showed 71.9% of respondents indicated consumption of contaminated water as a cholera transmission mode while 86.0% identified hand washing as a preventive measure [24]. In cholera endemic regions, rates of high knowledge on cholera from survey data range from 46.0% in Bangladesh to 84.8% in Tanzania [20], [21].

This study demonstrates that an OCV campaign with a strong public health education component was associated with increase in knowledge of cholera transmission, preventive measures, and methods of treating water. We also observed significant improvement in health practices essential for prevention of waterborne diseases after the vaccine campaign. Beau de Rochars et al. similarly reported significant improvement in water treatment practices in Haiti from 30.3% before cholera to 73.9% after community wide education campaigns in response to the outbreak [24]. Currently, there are no available data on the impact of a cholera vaccine program on knowledge and behavior related to cholera. Our cholera vaccination campaign provided an opportunity to raise awareness and directly reinforce public health messages about cholera control in the target population. Our findings demonstrate that an OCV campaign can be complementary to and even strengthen other cholera control efforts during an epidemic. Similarly, other vaccination programs may potentially function as health system strengthening tools in resource limited settings.

Our study also showed an association between knowledge and practice. Although a KAP study in Bangladesh demonstrated that good knowledge of cholera was associated with better prevention practices [20], other studies have shown hygiene practice rates were not commensurate with knowledge [21], [23]. It is important to note that KAP surveys do not explore the nuances of the social and economic context that influence or even deter the translation of knowledge into practice. For example, our surveys identified access to products as an important barrier to the practice of frequent water treatment. We also found that surrogates of higher socioeconomic status were associated with increased frequency of hand washing and water treatment. This may be attributed to the fact that individuals of higher socioeconomic status are likely able to afford soap and products for treating water. Although these products are distributed periodically free of charge by government and non-government organizations, they ordinarily must be purchased. They were not distributed to households at the time of the survey, but distributions did take place to some extent between the two surveys. Despite the apparent association between knowledge and practice, it is crucial to consider the various factors beyond information that influence health practices, particularly in resource limited settings. Moreover, it is not yet evident how levels of knowledge and hygiene practices as measured by KAP surveys actually impact cholera epidemics. To our knowledge, no data exists to confirm that higher knowledge and improved hygiene as measured by KAP surveys result in improved outcomes (e.g. decreased incident cases and mortality rates) in areas experiencing an epidemic.

This study has some limitations. First, we cannot exclude that other factors or interventions, external to the OCV campaign, were responsible for the findings. Nevertheless, despite our organization's presence in the area, work with the Ministry of Health, and consultation with the local water authorities at the time of writing, we are unaware of any other blanket community hygiene and education programs that occurred between the two surveys, other than our OCV campaign activities and routine public health messaging about the epidemic. There are technical water improvement initiatives that began in April 2012, but they do not have significant community educational components related to cholera or waterborne disease. A pre and post survey outside the catchment area of the OCV campaign would have provided comparison data, but this was not feasible as part of the vaccination campaign. Second, our study relied on self-report to assess water treatment and other hygiene practices so we cannot verify that reported practice was actual practice. Third, while we aimed for random, systematic sampling, the programmatic nature of the survey and the environment presented challenges in its execution. In Survey1, we interviewed 8.5% of households that completed the census, which was lower than 10% that would be expected when surveying every 10^th^ household. If some enumerators restarted their count of every 10^th^ household daily, instead of continuing the count across the days over the two-week census, this would explain the lower than expected survey rate. For post-vaccine surveys, we were unable to survey 13.7% of the 600 randomly generated households, partly due to lack of visible address markings on homes, families who moved away, lack of directions for homes in the census data, and challenges related to the rainy season. We lack information to assess whether households surveyed and not surveyed were comparable and whether respondents were similar across the two surveys. Nonetheless, we believe that it is unlikely there was a systematic bias in the inclusion households, and therefore it is unlikely that excluded households had significantly better or worse knowledge and practice about cholera than the included households. If surveyed households had different knowledge levels and practices than those not surveyed, this would bias our absolute estimates of knowledge and practice, but would unlikely influence our overall findings of improved knowledge and practice unless the extent or pattern of excluded households differed across the two Surveys. Finally the unequal distribution of some sociodemographic characteristics between the two survey populations raises the possibility of unmeasured differences in populations sampled. However, we believe that the observed differences reflect population-level changes over time such as seasonal variations in occupation and latrine access. For instance, post-vaccine surveys were administered later in the agricultural season when more participants may have identified as being farmers. Latrines are also at increased risk of overflowing in the rainy season, thus potentially forcing more individuals to resort to open defecation.

### Conclusion

After an integrated cholera vaccination campaign in rural Haiti, surveys demonstrate a significant increase in knowledge of cholera transmission and prevention mechanism as well as improvement in practices of water treatment and frequent hand washing, which are critical for curbing the spread of diarrheal diseases such as cholera. This provides evidence that oral cholera vaccination can be part of comprehensive cholera control and can reinforce, rather than detract from, other prevention activities in Haiti.

## Supporting Information

Figure S1 **Country map indicating location of an oral cholera vaccination campaign and knowledge and practice surveys in rural Haiti (Bocozel), 2012.**
(TIF)

Table S1 **Key education messages.** Key education messages before and during a cholera vaccination campaign in rural Haiti, 2012 (translated from Haitian Creole).
(DOC)
