# Correction: Cholera Vaccination Campaign Contributes to Improved Knowledge Regarding Cholera and Improved Practice Relevant to Waterborne Disease in Rural Haiti

**DOI:** 10.1371/annotation/13f018ff-b71a-4c5e-a276-d4b4ae608fee

**Published:** 2013-12-13

**Authors:** Omowunmi Aibana, Molly F. Franke, Jessica E. Teng, Johanne Hilaire, Max Raymond, Louise C. Ivers

There is an error in the names of the second and third authors. The corrected names are Molly F. Franke and Jessica E. Teng, respectively. The citation should read as follows:

Aibana O, Franke MF, Teng JE, Hilaire J, Raymond M, et al. (2013) Cholera Vaccination Campaign Contributes to Improved Knowledge Regarding Cholera and Improved Practice Relevant to Waterborne Disease in Rural Haiti. PLoS Negl Trop Dis 7(11): e2576. doi:10.1371/journal.pntd.0002576 

