# Supplementary figures and images for: Cholera Vaccination Campaign Contributes to Improved Knowledge Regarding Cholera and Improved Practice Relevant to Waterborne Disease in Rural Haiti
Source: PLoS Negl Trop Dis. 2013 Nov 21;7(11):e2576. doi: 10.1371/journal.pntd.0002576 (PMC3837010; doi:10.1371/journal.pntd.0002576)

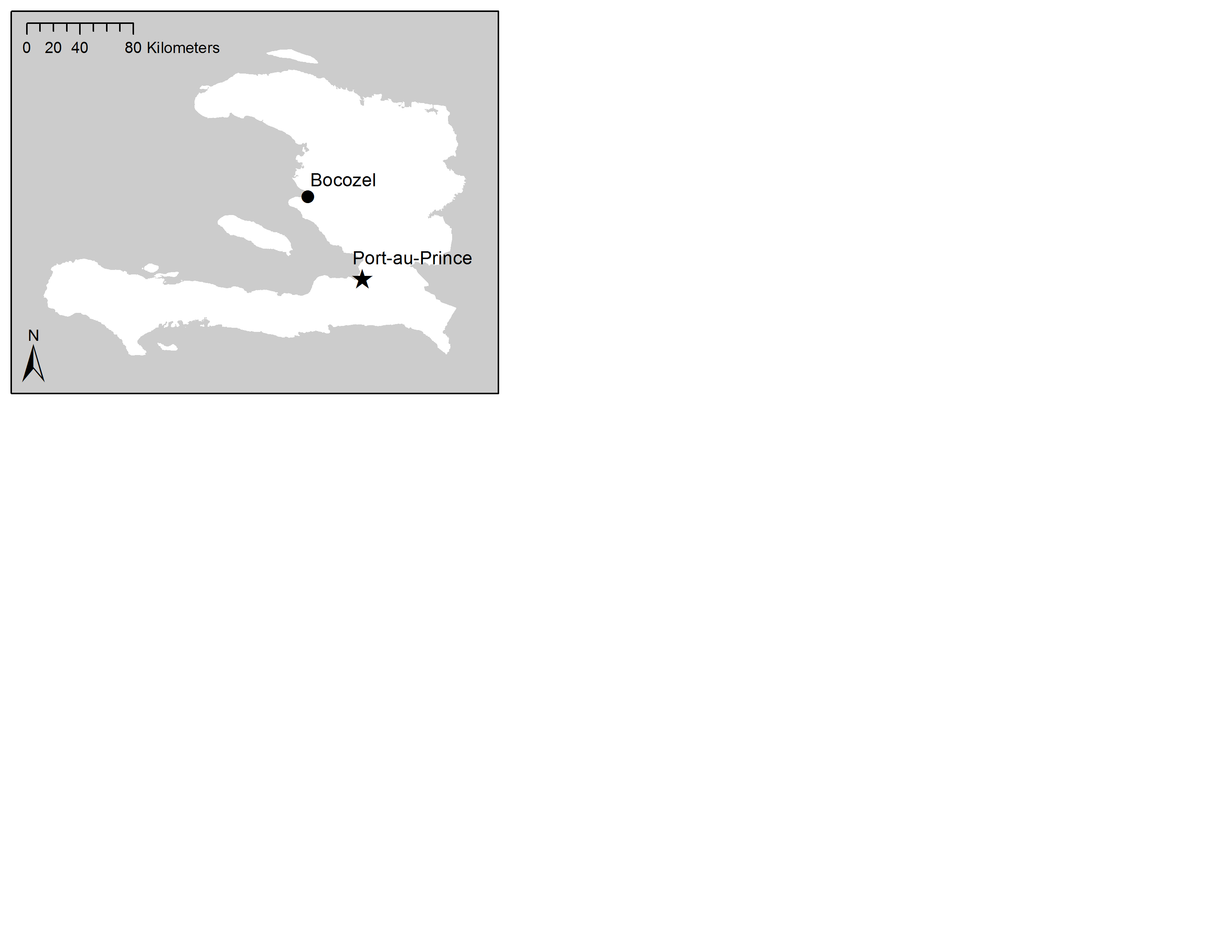

Supplement: Figure S1 — Country map indicating location of an oral cholera vaccination campaign and knowledge and practice surveys in rural Haiti (Bocozel), 2012. (TIF) [file pntd.0002576.s001.tif]
