# Supplementary material for: Cholera Vaccination Campaign Contributes to Improved Knowledge Regarding Cholera and Improved Practice Relevant to Waterborne Disease in Rural Haiti
Source: PLoS Negl Trop Dis. 2013 Nov 21;7(11):e2576. doi: 10.1371/journal.pntd.0002576 (PMC3837010; doi:10.1371/journal.pntd.0002576)
Supplement: Table S1 — Key education messages. Key education messages before and during a cholera vaccination campaign in rural Haiti, 2012 (translated from Haitian Creole). (DOC) [file pntd.0002576.s002.doc]

**Table S1: Key education messages before and during a cholera vaccination campaign in rural Haiti, 2012 (translated from Haitian Creole)**

| 1 | Cholera vaccine, when used in addition to hygiene measures, can help protect you against the cholera epidemic. |
| --- | --- |
| 2 | Cholera vaccine is given orally in 2 doses. It is administered by mouth in the same way as they give some childhood vaccines. |
| 3 | After taking the first dose, you must come back for the second dose in two weeks. If you do not receive the second dose, you will not get the best benefit of the vaccine. |
| 4 | Children less than 1 year and pregnant women cannot take the vaccine. |
| 5 | Even if you receive the cholera vaccine, you should always follow other hygiene principles, like washing your hands after you eat and use the toilet. You should always drink treated water. |
| 6 | Remember that cholera is not the only disease that causes diarrhea. Therefore, you should continue to respect all hygiene measures and keep your hands clean to avoid diarrhea. |
| 7 | Even if you receive cholera vaccine, you should keep oral rehydration solution in your house. If you develop diarrhea, drink the solution and go to the [nearest health post] to seek necessary care. |
| 8 | Children who receive cholera vaccine should get all other vaccines according to the normal schedule recommended by the Ministry of Health, including the vaccines that will be given in the upcoming months. |
| 9 | Remember that everyone is vulnerable to getting cholera. Let’s have a successful vaccination campaign and try to get some relief from the cholera epidemic. |
